# Bacterial inhibition potential of 3D rapid-prototyped magnesium-based porous composite scaffolds–an *in vitro* efficacy study

**DOI:** 10.1038/srep13775

**Published:** 2015-09-08

**Authors:** Rui Ma, Yu-xiao Lai, Long Li, Hong-lue Tan, Jia-li Wang, Ye Li, Ting-ting Tang, Ling Qin

**Affiliations:** 1 Shanghai Key Laboratory of Orthopedic Implants, Department of Orthopedic Surgery, Shanghai Ninth People’s Hospital, Shanghai Jiao Tong University School of Medicine, Shanghai, China; 2 Translational Medicine Research and Development Centre, Institute of Biomedical and Health Engineering, Shenzhen Institute of Advanced Technology, The Chinese Academy of Sciences, Shenzhen, China; 3 Department of Orthopedics and Traumatology, The Chinese University of Hong Kong, Hong Kong, China; 4 Department of Orthopedic Surgery, the Second Affiliated Hospital of Xi’an Jiaotong University, Xi’an, Shanxi Province, China

## Abstract

Bone infections are common in trauma-induced open fractures with bone defects. Therefore, developing anti-infection scaffolds for repairing bone defects is desirable. This study develoepd novel Mg-based porous composite scaffolds with a basal matrix composed of poly(lactic-co-glycolicacid) (PLGA) and tricalcium phosphate (TCP). A unique low-temperature rapid prototyping technology was used to fabricate the scaffolds, including PLGA/TCP (PT), PLGA/TCP/5%Mg (PT5M), PLGA/TCP/10%Mg (PT10M), and PLGA/TCP/15%Mg (PT15M). The bacterial adhesion and biofilm formation of Staphylococcus aureus were evaluated. The results indicated that the Mg-based scaffolds significantly inhibited bacterial adhesion and biofilm formation compared to PT, and the PT10M and PT15M exhibited significantly stronger anti-biofilm ability than PT5M. *In vitro* degratation tests revealed that the degradation of the Mg-based scaffolds caused an increase of pH, Mg^2+^ concentration and osmolality, and the increased pH may be one of the major contributing factors to the antibacterial function of the Mg-based scaffolds. Additionally, the PT15M exhibited an inhibitory effect on cell adhesion and proliferation of MC3T3-E1 cells. In conclusion, the PLGA/TCP/Mg scaffolds could inhibit bacterial adhesion and biofilm formation, and the PT10M scaffold was considered to be an effective composition with considerable antibacterial ability and good cytocompatibility.

Repairing bone defects is a formidable challenge in orthopedic clinics, because osteogenesis and angiogenesis in the defect sites are difficult and infections are often involved^1^. To some extent, the implant materials or grafting bones are the carriers for bacterial growth^2^. Bacteria can form biofilms on an abiotic surface, which is considered to be the key step during the occurrence of infection that prevents the healing process^3^.

Various bone substitutes have been developed for bone defect repair applications, including biodegradable polymers, bioceramics, and polymer-bioceramic composites. Biodegradable polymers, such as polylactic acid (PLA), polyglycolic acid (PGA), poly(lactic-co-glycolic acid) (PLGA), and poly-ε-caprolactone (PCL), can gradually be replaced by new bone, but their mechanical strength is too low^4^, and their degradation products may result in some side effects^5^. Bioceramic materials, such as hydroxyapatite (HA), tricalcium phosphate (TCP), and bioglasses, are all bioactive and offer good osteoconductivity; however, their application is generally limited due to their intrinsic brittleness and low fracture strength^6^. Composites composed of biodegradable polymers and bioceramics, such as PLGA/bioglass^7^, PLA/HA^8^, and PCL/HA^9^, can benefit from the advantages of both phases and can be tailored to mimic the native bone structure^10^,^11^. However, the majority of bone substitutes focus on bone repair only in a sterile environment, without considering how to manage the prevention and treatment of infection. Therefore, developing novel bone substitutes with the ability to both repair bone and prevent infections is desirable for conditions with a high risk of infection.

Some biometals have been reported to exhibit antibacterial activity, such as silver (Ag), zinc (Zn), and copper (Cu). The antibacterial activity of Ag was first demonstrated in the 19th century^12^, and Ag has been evaluated as an antimicrobial coating on orthopedic devices^13^,^14^. One significant problem with the use of Ag is that it can be toxic to host cells and tissues^13^,^15^. Zn^16^,^17^ and Cu^18^,^19^ have also been reported to possess antibacterial activity, but both of these metals have the potential for toxic sequelae. Magnesium (Mg) is an inexpensive and readily available metal, and it is abundant in bone and essential to many processes in eukaryotic cells^20^,^21^,^22^. Mg and Mg alloys have been developed for orthopedic^20^,^23^,^24^, cardiovascular^25^, and ureteral stent applications^26^ because of their good biocompatibility, unique biodegradability and satisfactory mechanical properties^27^. Our recent studies have evaluated the potential of Mg-based alloys for orthopedic applications^28^,^29^,^30^,^31^. Recent studies^32^,^33^ have reported that Mg also possesses antibacterial potential due to the increase of pH that occurs during its degradation.

Although a variety of conventional scaffold fabrication techniques are available, most are limited by some flaws, including manual intervention, shape limitations, inconsistent and inflexible processing procedures, and the use of toxic organic solvents and porogens^34^. Rapid prototyping (RP) technology is an emerging technology that can quickly create highly complex 3D models using medical imaging systems, computer-aided design (CAD) and digital converters, and it can precisely control the molding of different parts^35^,^36^. Low-temperature deposition manufacturing (LDM) is a unique RP technology that provides accurate point-to-point control of liquid molding materials, extrusion or injection at low temperature, rapid solidification, phase separation to form micropores, freeze-drying and solvent evaporation to form scaffolds with a gradient pore structure^37^. In our previous studies, we successfully fabricated a porous PLGA/TCP scaffold using the LDM technique, and we demonstrated that the PLGA/TCP scaffold exhibited good bone-repairing ability and can also be used as a drug carrier^38^,^39^,^40^,^41^.

The present study was designed to incorporate different concentrations of Mg into the PLGA/TCP matrix to fabricate porous PLGA/TCP/Mg composite scaffolds using the LDM technique. The antibacterial activity and cytocompatibility of these novel Mg-based scaffolds were investigated and compared with PLGA/TCP as a control. The mechanism for the antibacterial ability of these Mg-based scaffolds was also investigated.

## Results

### Bacterial adhesion assay

**Figure 1:**
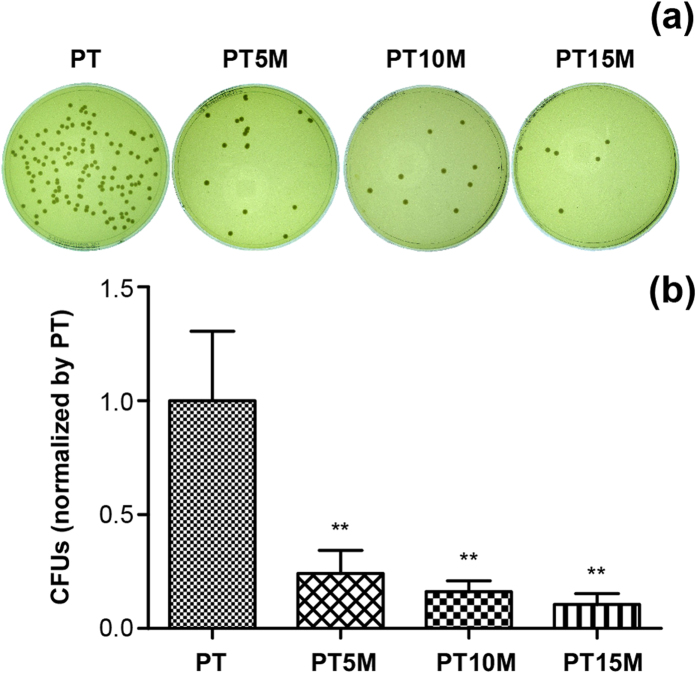
Bacterial adhesion at 6 hours as determined using the spread plate method: (**a**) Representative images of TSA with bacterial colonies from adhered bacteria; (**b**) Quantitative analysis of bacterial adhesion. The numbers of viable bacteria were counted and normalized to the counts from the PT group. ** denotes a significant difference compared to the PT group (p < 0.01).

Figure 1 presents the results of the bacterial adhesion assay using the spread plate method. Colonies were found less in the Mg groups (PT5M, PT10M, and PT15M) compared with the PT group (Fig. 1a). The bacterial adhesion was quantitatively determined by counting the numbers of colonies on TSA, as shown in Fig. 1b. The CFUs on PT5M, PT10M and PT15M (normalized by PT) were 24.2%, 16.1% and 10.6%, respectively, which are significantly less than that of PT (*p* < 0.01 for all). With respect to PT, the bacterial on PT5M, PT10M and PT15M reduced 0.62-log, 0.79-log and 0.97-log, respectively (Table 1).

### Bacterial growth assay

**Figure 2:**
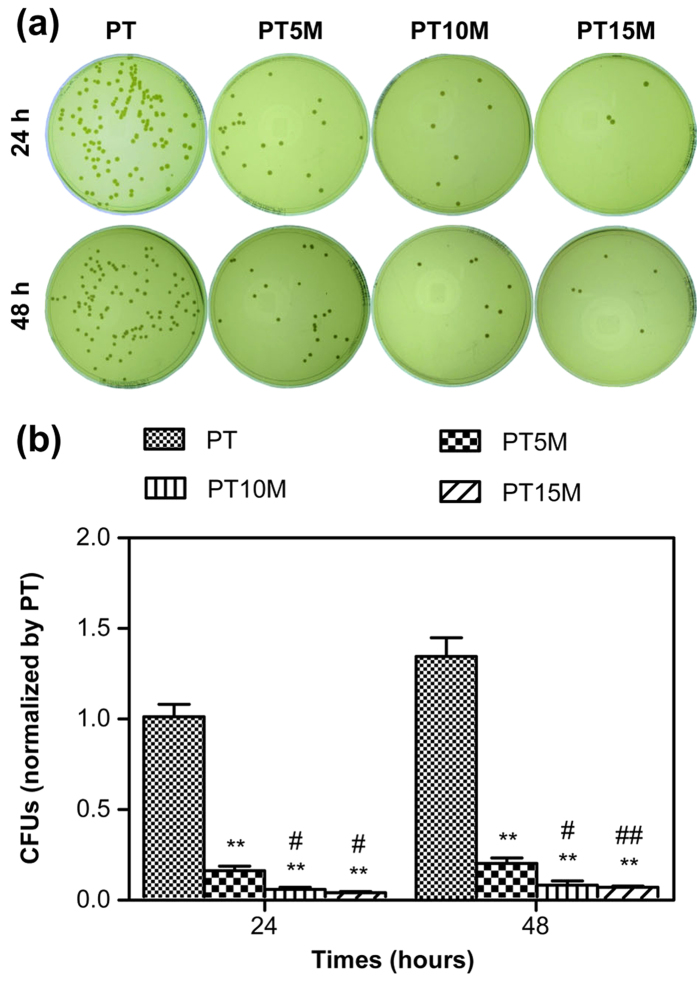
The number of viable bacteria on the surface of different specimens at 24 and 48 hours as determined using the spread plate method: (**a**) Representative images of TSA with bacterial colonies on the surfaces of different specimens; (**b**) Quantitative analysis of viable bacteria. The numbers of colonies were counted and normalized to the counts from the PT group at 24 hours. **denotes a significant difference compared to the PT group (p < 0.01); #denotes a significant difference compared to the PT5M group (#p < 0.05; ##p < 0.01).

Figure 2 presents the results of the bacterial growth assay using the spread plate method. As shown in Fig. 2a, there was a small number of colonies on the Mg groups (PT5M, PT10M, and PT15M) at 24 and 48 hours; in contrast, a large number of colonies was present on PT. Quantitatively, the CFUs of the Mg groups were significantly less than that of the PT group at each time point (*p* < 0.01, Fig. 2b). Considering log-reduction with respect to PT, the bacteria on PT, PT5M and PT15M reduced 0.95-log, 1.45-log and 1.65-log at 24 hours, while at 48 hours the bacteria on PT, PT5M and PT15M reduced 0.72-log, 1.15-log and 1.22-log, respectively (Table 1). Comparing between the three Mg groups, the numbers of colonies on the PT10M and PT15M groups were significantly lower than that on the PT5M group at 24 and 48 hours (*p* < 0.05, Fig. 2b).

**Figure 3:**
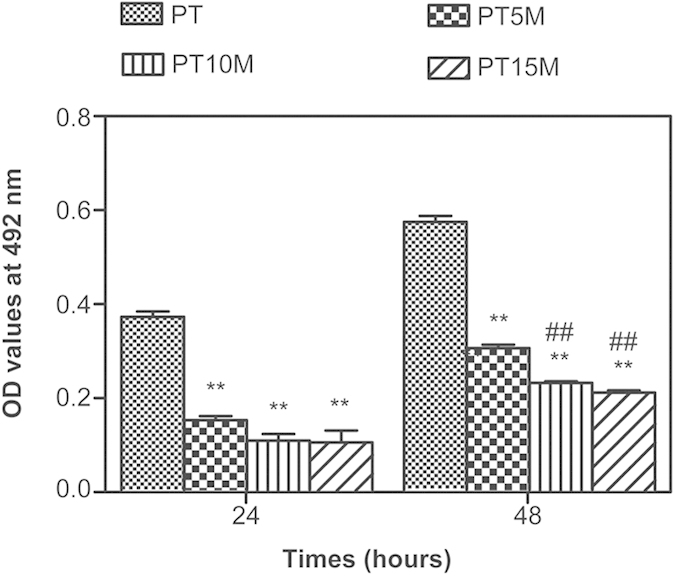
Biofilm formation assay as determined using the TCP method. **denotes a significant difference compared to the PT group (p < 0.01); ##denotes a significant difference compared to the PT5M group (p < 0.01).

The results of biofilm formation assessed using the TCP method are presented in Fig. 3. The OD values of the PT5M, PT10M and PT15M groups were significantly lower than that of the PT group at 24 and 48 hours (*p* < 0.01). The OD values of the PT10M and PT15M groups were clearly lower than that of the PT5M group at 48 hours (*p* < 0.01), which was consistent with the results determined using the spread plate method (Fig. 2b). In addition, no difference was observed between the PT10M group and the PT15M group at each time point (*p* > 0.05).

### SEM observation

**Figure 4:**
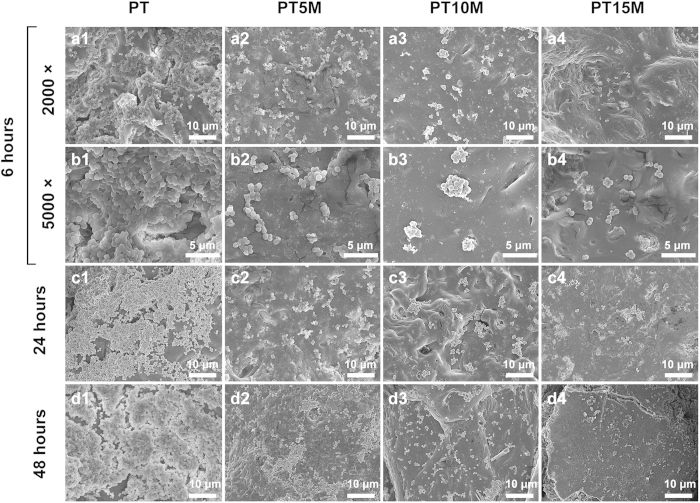
Representative SEM images showing bacterial adhesion and biofilm formation on surfaces of specimens.

As shown in Fig. 4, any adherent bacteria or formed biofilms could be observed in the SEM images. At 6 hours, there was a mass of clustered bacteria in the PT group (Fig. 4a1,b1); in comparison, fewer bacteria were observed in the PT5M group (Fig. 4a2,b2), PT10M group (Fig. 4a3), and PT15M group (Fig. 4a4,b4) relative to the PT group. After incubation for 24 hours, fewer scattered single colonies were observed in the PT5M (Fig. 4c2), PT10M (Fig. 4c3) and PT15M (Fig. 4c4) groups, particularly in the PT15M group; however, a biofilm was observed, which was formed by a large number of multiple bacteria colonies in the PT group (Fig. 4c1). At 48 hours, a thick layer of biofilm formed in the PT group (Fig. 4d1), and some large clustered bacterial colonies formed in the PT5M group (Fig. 4d2); in contrast, a few disperse bacterial colonies were present in the PT10M (Fig. 4d3) and PT15M groups (Fig. 4d4). As the incubation time increased (from 6 to 48 hours), bacteria increasingly grew on the PT surface with an apparent biofilm formed at 24 and 48 hours, but this trend was not apparent in the PT10M and PT15M groups.

### CLSM observation

**Figure 5:**
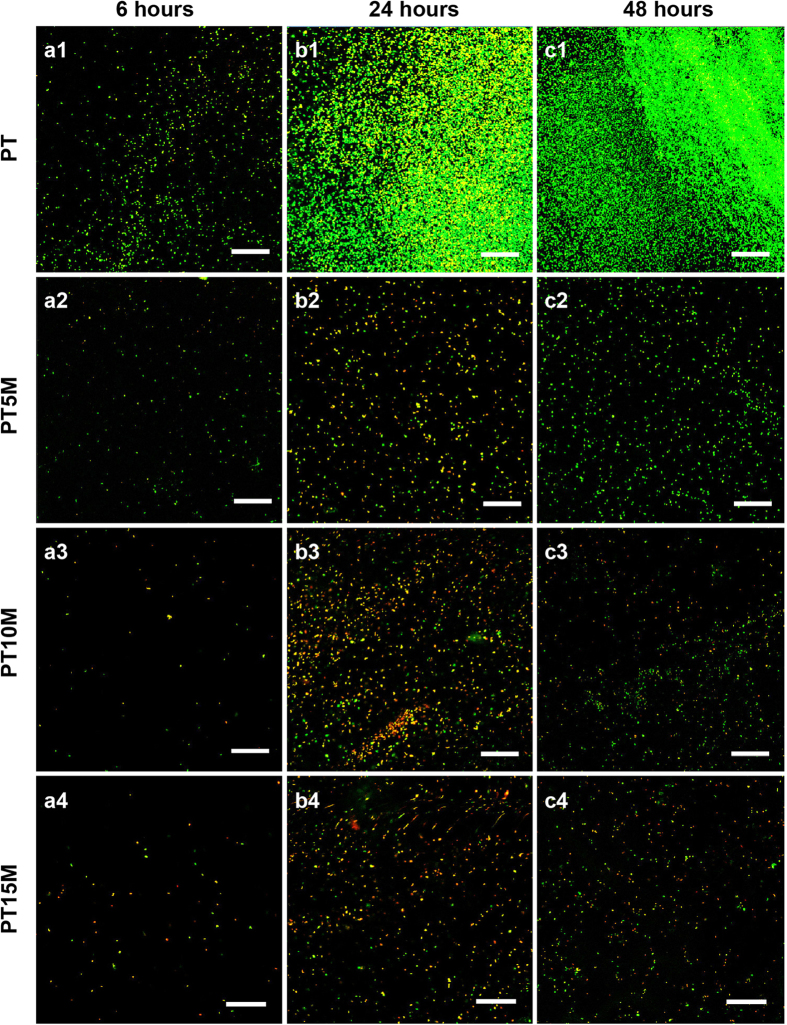
CLSM images showing live and dead bacteria in different groups. Live cells appeared green and dead cells appeared red under CLSM. Scale bar is 50 μm.

In Fig. 5, which presents CLSM images, live bacteria appeared with green fluorescence and dead ones appeared with red fluorescence. At 6 hours, considerably less green fluorescence could be observed on the PT5M, PT10M and PT15M groups (Fig. 5a2,a3,a4) than on the PT group (Fig. 5a1), indicating that less live bacteria adhered on PT5M, PT10M and PT15M than on PT. An extraordinarily dense green fluorescence indicating bacterial colonization could be observed on the PT group at 24 (Fig. 5b1) and 48 hours (Fig. 5c1). However, the green fluorescence was dispersed on PT5M, PT10M and PT15M (Fig. 5b2–b4,c2–c4), indicating no biofilm formation. Comparing the fluorescence denseness of the three Mg groups (PT5M, PT10M and PT15M), it could be observed that the red fluorescence was denser on PT10M and PT15M than on PT5M at 24 and 48 hours, and the green fluorescence was sparser on PT10M and PT15M than on PT5M at 48 hours. In other words, more dead bacteria and less live bacteria were present on PT10M and PT15M than on PT5M at 24 and 48 hours.

### *In vitro* degradation

**Figure 6:**
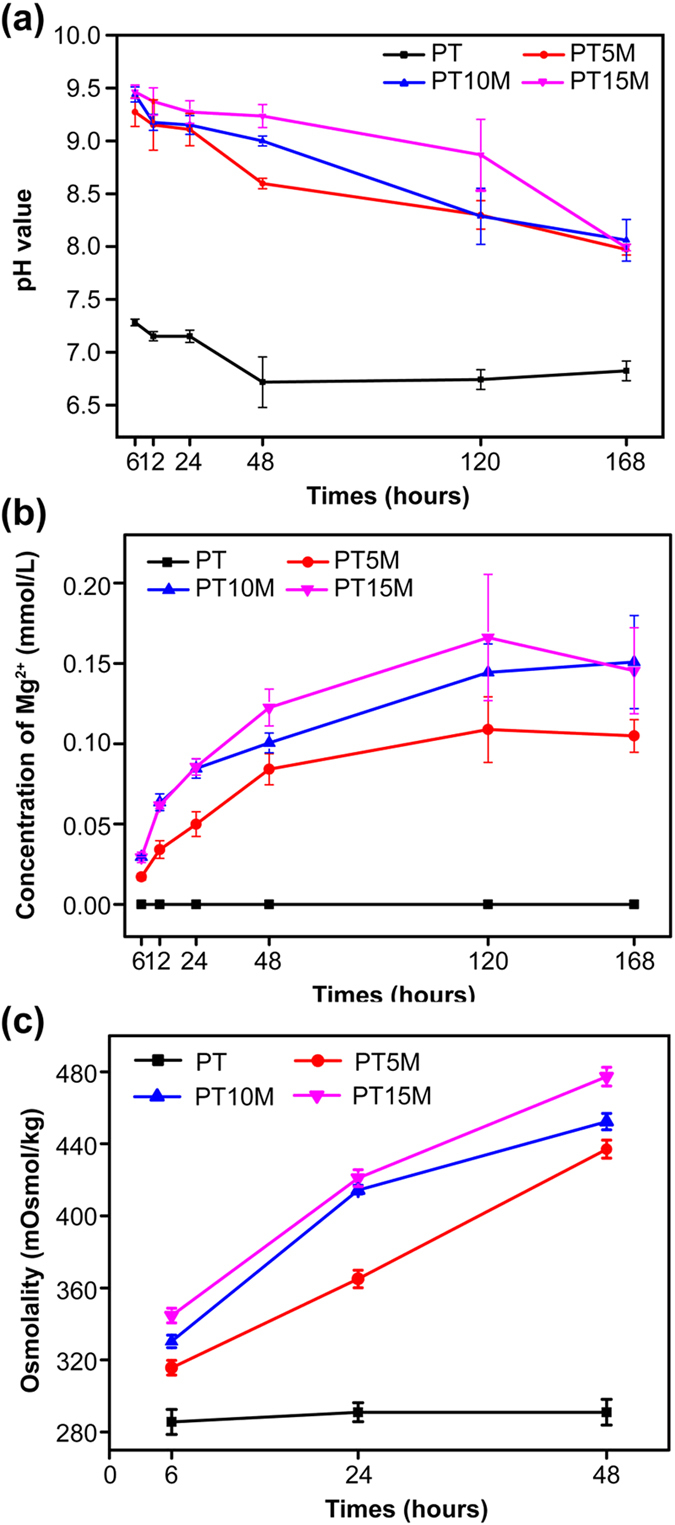
Degradation features of the PLGA/TCP/Mg scaffolds: (**a**) Changes in pH of the degradation medium; (**b**) Changes in ionic concentration of Mg^2+^; (**c**) Changes in osmolality.

The results of the *in vitro* degradation tests are shown in Fig. 6. The pH of the PT group was less than 7.4 along with the degradation of the PT scaffold within 168 hours (Fig. 6a). In contrast, the pH values of all three Mg groups (PT5M, PT10M, and PT15M) were greater than 7.4 within 168 hours, greater than 8.5 within 48 hours, and greater than 9 within 24 hours. The pH of PT15M was obviously higher than those of PT10M at 12, 48, and 120 hours (p < 0.05) and higher than those of PT5M at each time point within 120 hours (p < 0.05). The pH values of PT10M was clearly higher than those of PT5M at 6 and 48 hours (p < 0.05). All Mg groups released Mg^2+^ into the extracts, leading to a gradual increase in the Mg^2+^ concentration within 168 hours (Fig. 6b). The degradation of the PT group caused no changes in the osmolality (Fig. 6c). However, the osmolalities of the three Mg groups increased within 48 hours, and comparison among them showed PT15M > PT10M > PT5M at each time point (p < 0.05).

### Mechanism for the antibacterial activity of the Mg-based scaffolds

**Figure 7:**
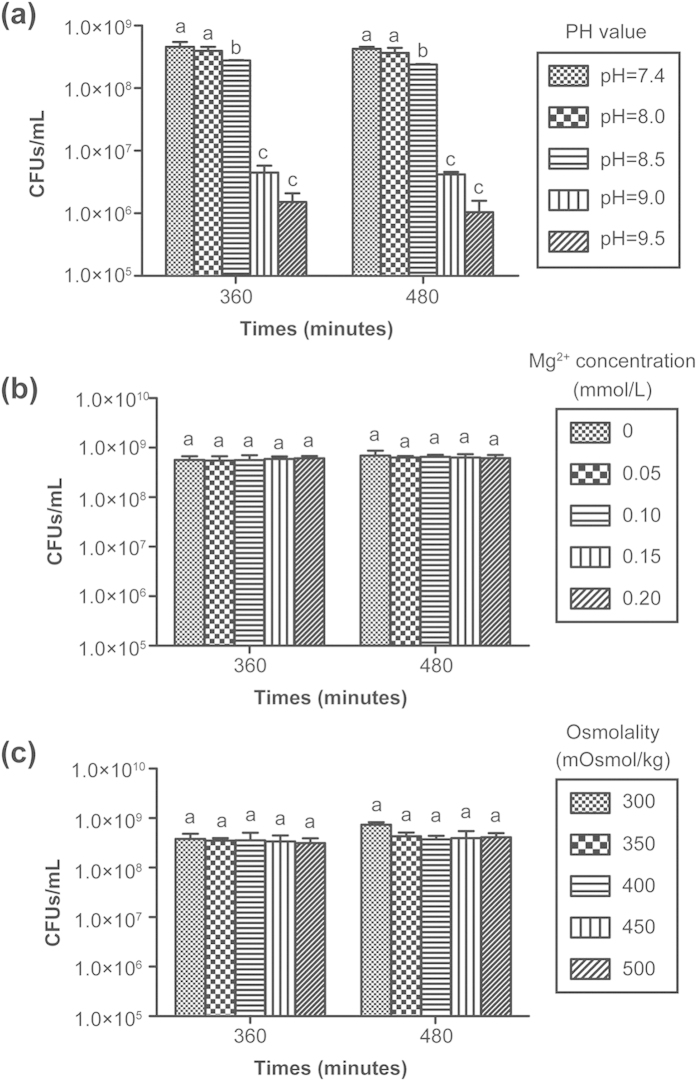
The number of viable bacteria from culture plates treated using different pH values (**a**), Mg^2+^ concentrations (**b**), and osmolality values (**c**). At each time point, columns labelled with the same letter are not significantly different (p > 0.05) but columns labelled with different letters are significantly different (p < 0.05).

Figure 7 presents the results of the effect of different pH values, Mg ion concentrations, and osmolality values on the bacterial activity. Figure 7a shows that different pH values had an influence on the bacterial activity. When the pH was 8.5, the number of bacteria clearly decreased compared to pH 7.4 or 8.0 (*p* < 0.05); when the pH reached 9.0 or 9.5, the anti-bacterial effect was more pronounced compared to pH 7.4, 8.0, or 8.5 (*p* < 0.01). Figure 7b shows that there was no significant difference between different ionic concentrations of Mg (*p* > 0.05), implying that a certain range of ionic concentrations of Mg (0–0.20 mmol/L) did not affect the vitality of bacteria. Similarly, no significant difference was found between different osmolalities (*p* > 0.05)(Fig. 7c), indicating that osmolality within a certain range (less than 500 mOsmol/kg) had no influence on the bacterial activity.

### *In vitro* cytocompatibility

**Figure 8:**
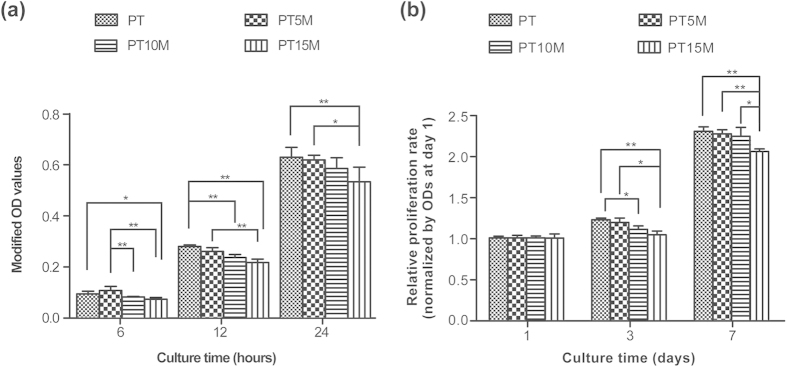
Cell adhesion and proliferation of MC3T3-E1 cells on the scaffolds: (**a**) Cell adhesion at 6, 12 and 24 hours; (**b**) Cell proliferation at 1, 3 and 7 days. Modified OD values were ODs at 450 nm subtracted by ODs at 620 nm. The modified OD values at 3 and 7 days were normalized to those at 1 day. * and ** denote p < 0.05 and p < 0.01, respectively.

The results of the *in vitro* cytocompatibility tests are shown in Fig. 8. The modified OD values represent the numbers of adherent cells on the specimen surface. The numbers of adherent cells on PT15M were significantly less than those on PT and PT5M at each time point (Fig. 8a, *p* < 0.05), indicating that cell adhesion on PT15M was unsatisfactory. The numbers of adherent cells on PT10M were less than those on PT5M at 6 hours and on PT at 12 hours (*p* < 0.05). Figure 8b shows that the MC3T3-E1 cells on PT15M exhibited a lower relative proliferation rate than those on PT and PT5M at 3 and 7 days (*p* < 0.05). The relative proliferation rate of cells on PT10M was lower than that on PT at 3days (*p* < 0.05), but higher than that on PT15M at 7 days (*p* < 0.05).

## Discussion

In this study, porous PLGA/TCP/Mg composite scaffolds were fabricated using the low-temperature RP technique. A number of studies have shown the benefits of rapid-prototyped composite scaffolds with PLGA/TCP^42^,^43^,^44^. In this composite system, PLGA and TCP are the basic scaffold matrix materials that can compensate each other in terms of their physical or mechanical properties, i.e., with the good stiffness of TCP and the flexibility of PLGA to form a composite scaffold of PLGA/TCP. In addition, the alkaline degradation product of TCP can neutralize the acidic degradation products of PLGA. More importantly, the introduction of Mg may impart potential antibacterial activity. Our research teamhas performed a systematic evaluation of the antibacterial properties of pure magnesium^45^. The results demonstrated that Mg reduced bacterial adhesion and prevented biofilm formation *in vitro*, and protected the implant from bacterial contamination and improved new peri-implant bone formation *in vivo*. Using different methods to assess the antibacterial potential of the prepared Mg-based scaffolds, we found that the Mg-based composite scaffolds significantly inhibited bacteria adhesion and biofilm formation of Staphylococcus aureus on their surfaces.

After a material is implanted, osteoblasts and bacteria will compete with each other to adhere to the surface of the material^46^. If the adhesion of osteoblasts is faster than that of bacteria, bone matrix will gradually be deposited on the material; but it is not the case, infection may occur. It is more reasonable to conduct an *in vitro* competition experiment between osteoblast adhesion and bacterial adhesion. However, such co-culture system is challenging because the growth mediums for bacteria and for osteoblasts were different and the bacterial endotoxins were poisonous to human cells. Therefore, we conduct bacterial culture and cell culture separately. In our study, the Mg-based scaffolds clearly exhibited a strong ability to inhibit bacterial adhesion compared with the PLGA/TCP scaffold at 6 hours. This inhibiting effect may contribute to the corrosion of Mg, which produced an alkaline environment to inhibit the adhesion of bacteria.

The majority of bacteria that enter the bone defect site will colonize on the implant surface or necrotic tissue and produce many polysaccharide-protein complexes to wrap bacteria and form biofilms^47^. The ability of bacteria to develop antibiotic resistance and colonize abiotic surfaces by forming biofilms is a major cause of orthopedic infections^3^. The individual bacteria can be easily killed by the immune system or antibiotics *in vivo*, but killing bacteria in a biofilm is difficult because a biofilm has strong resistance to the host’s immune system and antibiotics^47^. It has been reported that the dose of an antibiotic required to kill the bacteria in a biofilm is approximately 1000 times greater than that required to kill planktonic bacteria^48^. Therefore, it is more important to prevent biofilm formation than to eliminate a biofilm. The ability to inhibit biofilm formation is crucial when evaluating the anti-infection activity of materials. Our results demonstrated that the Mg-based composite scaffolds could significantly inhibit biofilm formation at 24 and 48 hours, and PT15M and PT10M exhibited a stronger ability to inhibit biofilm formation than that of PT5M. However, further investigations are required to confirm whether even higher concentrations of Mg may have a stronger ability to inhibit biofilm formation.

Thus far, the mechanism of the Mg-based materials in inhibiting bacterial growth and biofilm formation is lacking. Robinson *et al.*^32^ considered that the degradable characteristic of Mg in a physiological solution could result in rapid increases in both the Mg^2+^ concentration and pH, and the latter should be responsible for the antibacterial function of Mg. Lock *et al.*^26^ reported that the degradation of Mg in artificial urine led to an increase in Mg ionic concentration and an increase in solution pH, both of which potentially contributed to the antibacterial property of the Mg-based materials. In our study, the prepared Mg-based scaffolds degraded to cause alkaline pH, increased Mg^2+^ concentration, and increased osmolality, all of which might be responsible for the antibacterial activity.

Most organisms have a pH range in which preferential growth occurs^49^. Bacteria can generally live in an environment with a pH range of 6.0–8.0, in which bacteria can maintain a cytoplasmic pH that is compatible with the optimal functional and structural integrity of the cytoplasmic proteins^50^. In our study, the Mg-based materials generated a higher pH, which was greater than 8.0 within 168 hours, and the highest pH was nearly 9.5 (Fig. 6a). The following experimental results demonstrated that a pH value that is greater than 8.5 had an inhibitory effect on bacterial vitality, particularly when the pH > 9.0 (Fig. 7a). These results suggested that the alkaline pH was at least one reason for the antibacterial ability and could explain why the anti-biofilm ability of PT10M and PT15M was stronger than that of PT5M.

Figure 7b illustrates whether the concentration of Mg ions plays a role in the observed antibacterial activity. The results indicated that the concentration of Mg ions provided no contribution to the antibacterial action. In Lock’s study^26^, they demonstrated a negative correlation between CFUs and magnesium ion concentration, and the increased magnesium ion concentrations were positively associated with the increase in pH of artificial urine solutions. They confirmed that the increased alkalinity of the solution inhibited bacterial growth, but the increased Mg ionic concentration was not confirmed to inhibit bacterial growth. The negative correlation between CFUs and magnesium ion concentration may have been indirectly correlated and mediated by the increased alkalinity.

The rapid changes caused by osmotic shock might lead to modifications of the phospholipid structure of the bacterial membrane and even bacterial death^51^,^52^. Although the degradation of the Mg-based scaffolds caused an increase inosmolality, our results revealed that the osmolality within a certain range between 0 and 500 mOsmol/kg did not influence bacterial activity. It is possible that the osmotic pressure between 0 and 500 mOsmol/kg caused by degradation of the Mg-based scaffolds is not sufficient to destroy the bacterial membrane. Considering these results and the published reports, the increased pH value caused by the degradation of Mg may be the primary cause for the antibacterial activity of Mg-based materials.

Biocompatibility is very important when evaluating a biomaterial. The prepared Mg-based scaffolds exhibited an increase in pH after Mg was degraded. However, an environment that is too alkaline will inhibit cell growth and even kill cells^53^. Our results demonstrated that PT15M was harmful to cell adhesion and proliferation because of the high pH in the culture medium caused by Mg corrosion. Considering the cytocompatibility of PT10M, cell adhesion was worse than that of PT5M at 6 hours and of PT at 12 hours, and cell proliferation was worse than that of PT at 3 days; however, cell adhesion at 24 hours and cell proliferation at 7 days were comparable with those of PT and PT5M, which demonstrated that the cytocompatibility of PT10M was acceptable. In addition, the pH of PT10M rapidly decreased after 48 hours, which is more favorable to cell proliferation than that before 48 hours. Therefore, the optimal content of Mg was considered to be 10% considering both antibacterial activity and cytocompatibility.

In conclusion, this *in vitro* study demonstrated for the first time that PLGA/TCP/Mg scaffolds prepared using a unique low-temperature rapid prototyping technique exhibited the ability to inhibit bacterial adhesion and biofilm formation. The composite containing 10% Mg was considered to be a promising bone substitute with both antibacterial ability and good cytocompatibility. The degradation of the Mg-based scaffolds led to increases in the pH, Mg^2+^ concentration and osmolality in the local environment and the alkaline pH may be the main cause of the antibacterial activity of the PLGA/TCP/Mg scaffolds.

## Materials and Methods

### Raw materials

PLGA [LA/GA = 75/25, average molecular weight ≈ 15.5 × 10^4^, viscosity = 1.72 dL/g] was purchased from Shandong Institute of Medical Instruments, China. TCP (particle size ≈ 50 μm) was purchased from Beijing Modern Orient Precise Chemical Articles Ltd., China. Mg (particle size ≈ 50 μm) was purchased from Shenzhen Tianyuan Magnesium Ltd., China. 1,4-Dioxane was purchased from Sinoparm Chemical Reagent Ltd.

### Preparation of scaffolds

**Figure 9:**
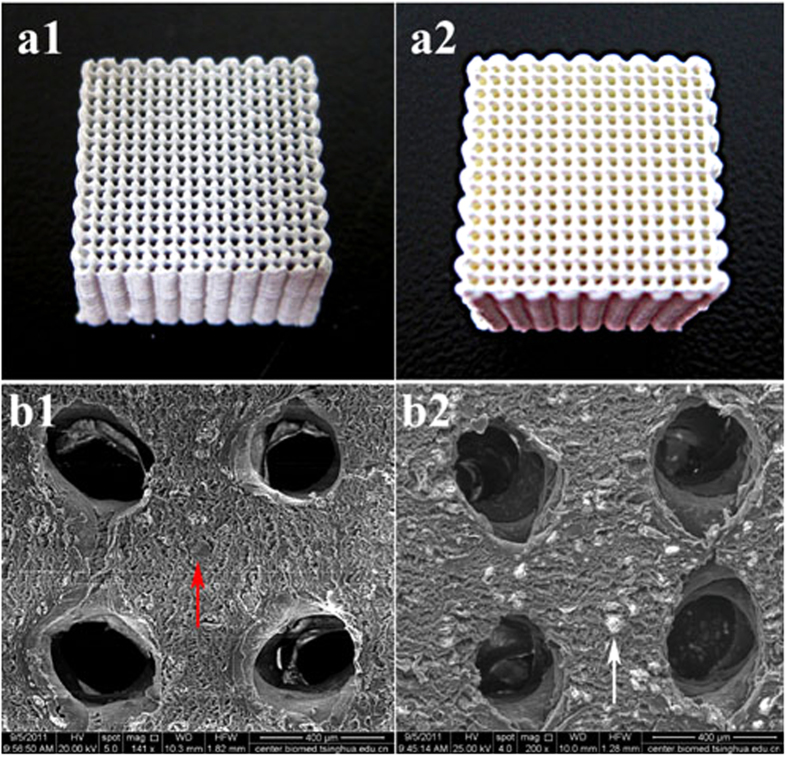
The pictures and SEM images of PLGA/TCP/Mg and PLGA/TCP scaffolds. a1, PT10M scaffold; a2, PT scaffold; b1, SEM image of PT10M scaffold, the red arrow indicated the Mg particle; b2, SEM image of PT scaffold, the white arrow indicated the TCP particle. Scale bar is 400 μm.

The porous PLGA/TCP/Mg composite scaffolds were fabricated according to our previously reportedprotocol^41^,^54^. The porous PLGA/TCP scaffolds were fabricated by low-temperature rapid prototyping technology so as for PLGA/TCP/Mg scaffold. Briefly, PLGA was dissolved in 1,4-Dioxane to form a homogeneous solution. TCP powders (PLGA/TCP (mass ratio) = 4:1) were then added to the PLGA solution. The porous PLGA/TCP scaffolds were fabricated at −30 °C using an advanced low-temperature rapid-prototyping machine. Three different scaffolds with different concentrations of Mg [PLGA/TCP/5 wt.% Mg (PT5M), PLGA/TCP/10 wt.% Mg (PT10M), and PLGA/TCP/15 wt.% Mg (PT15M)] were designed. The PLGA/TCP (PT) scaffold served as the control group. All of the porous scaffolds were spun layer-by-layer using a computer-driven nozzle according to the predesigned stereolithography model to form specific 3D porous scaffold blocks with a size of 10 × 10 × 10 mm^3^, which were then lyophilized. The picture and SEM image of the prepared PLGA/TCP/Mg and PLGA/TCP scaffolds were shown in Fig. 9.

### Preparation of bacteria

Staphylococcus aureus was used to evaluate the antibacterial activity in this study. American Type Culture Collection (ATCC) 25923 (Manassas, VA, USA) was purchased in freeze-dried form, and prepared using the plate streaking method.

### Quantitative analysis of bacterial adhesion and bacterial growth using the spread plate method

The spread plate method^55^ was used to quantitatively analyse the bacterial adhesion at 6 hours and bacterial growth at 24 and 48 hours. The inoculum of the strain was prepared by adjusting the concentration of an overnight bacterial broth culture to 1 × 10^6^ colony forming units (CFUs)/mL in TSB using McFarland standards (Beijing Zhecheng Science and Technology Co., Ltd., Beijing, China). A 500 μL aliquot of the suspension containing 1 × 10^6^ CFUs/mL bacteria was added to wells that contained specimens (three for each group) and incubated at 37 °C for 6 hours. Then, the specimens were gently washed three times with sterile phosphate-buffered saline (PBS) to remove the loosely adherent bacteria and then placed in 500 μL of TSB. The adherent bacteria on the specimens were removed by ultrasonication^56^. The ultrasonication was conducted in a 150 W, 50 Hz ultrasonic bath (B3500S-MT, Branson Ultrasonics Co., Shanghai, China) for 20 minutes. Then, the collected solutions after ultrasonication were subjected to a ten-fold dilution process. The 10^4^-, 10^5^-, and 10^6^-fold dilute solutions (100 μL) were plated onto TSA and then incubated at 37 °C for 24 hours. The number of colonies on the TSA was counted. The ultimate CFUs were the number of colonies multiplied by the dilution ratio. The CFUs of each group were normalized to the counts from the PT.

The bacterial growth assay was similar to the procedures used in the bacterial adhesion assay, except that the time points were 24 and 48 hours and the plated dilution ratios were 10^6^, 10^7^ and 10^8^. The CFUs of each group were normalized to the counts from the PT at 24 hours.

### Biofilm formation assay using the tissue culture plate (TCP) method

The TCP method is commonly used to quantitatively analyse biofilm formation^57^. The specimens with TSB served as the negative control. After 24 and 48 hours, the specimens were fixed with 2.5% glutaraldehyde for 30 minutes at 4 °C and dried at 60 °C for 1 hour. The biofilms were stained with 500 μL of 0.1% crystal violet (CV; Sigma-Aldrich, St. Louis, MO, USA) solution at room temperature for 20 minutes. The specimens were rinsed three times with PBS and dried at 37 °C for 2 hours. The stained CV was dissolved in 500 μL of 2% glacial acetic acid (Sigma-Aldrich) for 15 minutes with agitation at 200 rpm. The biofilms were quantified by the CV concentration, which was determined by measuring the optical density (OD) using a microplate reader (Synergy HT, Bio-tek, Winooski, VT, USA) at a wavelength of 492 nm. The mean OD obtained from the negative control was determined from the ODs of the test groups.

### Observation of bacterial adhesion and biofilm formation using scanning electron microscopy (SEM)

The specimens were incubated with 500 μL of bacterial suspensions of 1 × 10^6^ CFUs/mL in TSB for 6, 24, and 48 hours. At each time point, the specimens were gently washed three times with PBS, fixed in 2.5% glutaraldehyde at 4 °C for 30 minutes, washed three times with PBS again, and dehydrated with a series of graded ethanol solutions. Then, the specimens were air dried, sputter-coated with gold, and observed using a SEM (S-4800, Hitachi, Tokyo, Japan).

### Observation of bacterial adhesion and biofilm formation using confocal laser scanning microscopy (CLSM)

After 6, 24, and 48 hours, the specimens were stained with 300 μL of a combination dye (Live/Dead BacLight bacteria viability kits; Molecular Probes Life Technologies, Carlsbad, CA, USA) and observed with a CLSM (Leica TCS SP2, Heidelberg, Germany). Bacteria were stained with green fluorescent SYTO 9 and red fluorescent propidium iodide.

### *In vitro* degradation test

Culture medium extracts were first prepared by immersing pairs of scaffolds (0.3 g) in 3 mL of α-modified eagle’s medium (α-MEM; Hyclone, Thermo Fisher Scientific Inc., Miami, FL, USA) and incubated under 5% CO_2_ at 100% relative humidity for 6, 12, 24, 48, 72 and 168 hours. During this period, the pH values of the bulk solution were measured using a flat membrane microelectrode (PB-10, Sartorius, Germany). The ionic concentrations of Mg ions released from the scaffolds were estimated from scaffold extracts using an inductively coupled plasma optical emission spectrometer (ICP-OES, Optima 7000DV, PerkinElmer, Waltham, MA, USA). The osmolality values of the extracts prepared from the scaffolds after immersion in α-MEM for 6, 24 and 48 hours were measured using a vapor pressure osmometer (5520, Wescor, Logan, UT, USA)^58^. Five replicates were used for each group for data calculation.

### Mechanism analysis of the antibacterial activity of Mg-based scaffolds

The results of the *in vitro* degradation (Fig. 3) revealed that the degradation of the Mg-based scaffolds resulted in changes in three factors: increases in pH, Mg^2+^ concentration and osmolality. The following study was conducted to determine the main contributing factors to the potential antibacterial activity of the Mg-based scaffolds.

#### Adjustment of pH

Normal TSB medium was adjusted to have a pH of 7.4. TSB culture mediums with different pH values (8, 8.5, 9, and 9.5) were prepared by adding a 1 M NaOH solution dropwise to normal TSB while monitoring the mixture with a pH meter (Mettler Toledo320, Zurich, Switzerland). The Staphylococcus aureus (ATCC25923) bacteria were inoculated in TSB medium with different pH values (7.4, 8, 8.5, 9, and 9.5) in 48-well plates at a density of 1 × 10^6^ CFUs/mL. Then, the culture plates were incubated at 37 °C for 360 and 480 minutes. At each time point, the incubated bacterial suspensions were blended, plated onto TSA, incubated for 24 hours, and counted.

#### Adjustment of Mg^2+^ concentration

TSB media with different Mg^2+^ concentrations (0, 0.05, 0.10, 0.15, and 0.20 mmol/L) were prepared by adding MgCl_2_·6H_2_O to normal TSB medium. The effect of the Mg^2+^ concentration on the bacterial activity was investigated using the spread plate method.

#### Adjustment of osmolality

TSB media with different osmolalities (300, 350, 400, 350, and 400 mOsmol/kg) were prepared by adding a 5 M NaCl solution. The effect of the osmolality on the bacterial activity was also investigated using the spread plate method.

### *In vitro* cytocompatibility assay

MC3T3-E1 was used to investigate the *in vitro* cytocompatibilities of the scaffolds. The cells were cultured in α-MEM (Hyclone) supplemented with 10% fetal bovine serum (FBS; GibcoBRL, Grand Island, NY, USA) and 1% antibiotics (100 U/mL penicillin and 100 mg/mL streptomycin sulphate; GibcoBRL) at 37 °C in a humidified atmosphere with 5% CO_2_, with the culture medium changed every three days.

A cell counting kit-8 (CCK-8) assay was used to analyse cell adhesion on the specimens after 6, 12 and 24 hours. The MC3T3-E1 cells were seeded at a density of 6 × 10^4^/cm^2^ in a 48-well plate containing the specimens, with wells containing α-MEM as a negative control. The specimens and cells were co-incubated at 37 °C in a humidified atmosphere of 5% CO_2_. At each time point, a volume of 40 μL of CCK-8 solution (Dojindo Molecular Technologies Inc., Kumamoto, Japan) was added to each well and incubated for 3 hours at 37 °C. Then, the OD values were read at 450 nm and 620 nm using a microplate reader (Synergy HT, Bio-tek). The mean OD obtained from the negative control was subtracted from the ODs of the test groups. The cell proliferation was also investigated using the CCK-8 assay after 1, 3, and 7 days. The seeding density of the cells was 2 × 10^4^/cm^2^. The OD values at days 3 and 7 were normalized to those at day 1.

### Statistical analysis

All experiments were conducted in triplicate and repeated three times. The results were tested using one-way analysis of variance (ANOVA) and least significant difference (LSD) *post hoc* tests to determine any significances, with p < 0.05 being significant and *p* < 0.01 being highly significant. All statistical analyses were performed using SPSS software (version 13.0).

## Additional Information

**How to cite this article**: Ma, R. *et al.* Bacterial inhibition potential of 3D rapid-prototyped magnesium-based porous composite scaffolds – an *in vitro* efficacy study. *Sci. Rep.* **5**, 13775; doi: 10.1038/srep13775 (2015).

## Figures and Tables

**Table 1:** The log-reduction of bacteria on the PT5M, PT10M and PT 15M with respect to PT.

Times (hours)	Groups (–log)			
	PT	PT5M	T10M	PT15M
6	0	0.62	0.79	0.97
24	0	0.95	1.45	1.65
48	0	0.72	1.15	1.22
